# Fast Ultrasound Scanning is a Rapid, Sensitive, Precise and Cost-Effective Method to Monitor Tumor Grafts in Mice

**DOI:** 10.1007/s10911-024-09555-3

**Published:** 2024-01-30

**Authors:** Sébastien Molière, Arthur Martinet, Amélie Jaulin, Massimo Lodi, Thien-Nga Chamaraux-Tran, Fabien Alpy, Guillaume Bierry, Catherine Tomasetto

**Affiliations:** 1 Institute of Genetics and Molecular and Cellular Biology, Illkirch, France; 2 Centre National de la Recherche Scientifique, UMR 7104, Illkirch, France; 3 Institut National de la Santé et de la Recherche Médicale U1258, Illkirch, France; 4 University of Strasbourg, Strasbourg, France; 5 Department of Radiology, Strasbourg University Hospital, Hôpital de Hautepierre, Strasbourg, France; 6 Breast and Thyroid Imaging Unit, ICANS, Strasbourg, France; 7 Department of Anesthesiology, Groupe Hospitalier Saint Vincent, Clinique Sainte Barbe, Strasbourg, France; 8 Engineering Science, Computer Science and Imaging Laboratory (ICube), Integrative Multimodal Imaging in Healthcare, UMR 7357, University of Strasbourg-CNRS, Strasbourg, France

**Keywords:** Mouse, Tumor graft, Ultrasound, Bioluminescence, Breast tumor, Follow-up

## Abstract

In preclinical studies, accurate monitoring of tumor dynamics is crucial for understanding cancer biology and evaluating therapeutic interventions. Traditional methods like caliper measurements and bioluminescence imaging (BLI) have limitations, prompting the need for improved imaging techniques. This study introduces a fast-scan high-frequency ultrasound (HFUS) protocol for the longitudinal assessment of syngeneic breast tumor grafts in mice, comparing its performance with caliper, BLI measurements and with histological analysis. The E0771 mammary gland tumor cell line, engineered to express luciferase, was orthotopically grafted into immunocompetent C57BL/6 mice. Tumor growth was monitored longitudinally at multiple timepoints using caliper measurement, HFUS, and BLI, with the latter two modalities assessed against histopathological standards post-euthanasia. The HFUS protocol was designed for rapid, anesthesia-free scanning, focusing on volume estimation, echogenicity, and necrosis visualization. All mice developed tumors, only 20.6% were palpable at day 4. HFUS detected tumors as small as 2.2 mm in average diameter from day 4 post-implantation, with an average scanning duration of 47 s per mouse. It provided a more accurate volume assessment than caliper, with a lower average bias relative to reference tumor volume. HFUS also revealed tumor necrosis, correlating strongly with BLI in terms of tumor volume and cellularity. Notable discrepancies between HFUS and BLI growth rates were attributed to immune cell infiltration. The fast HFUS protocol enables precise and efficient tumor assessment in preclinical studies, offering significant advantages over traditional methods in terms of speed, accuracy, and animal welfare, aligning with the 3R principle in animal research.

## Introduction

Tumor grafts in preclinical models have become instrumental platforms for probing cancer dynamics, investigating tumor biology, deciphering intricate tumor-host interactions, and evaluating the potential of novel therapeutic interventions [1]. Consequently, a precise, continuous, and robust assessment of tumor growth and evolution is paramount in these models. Such evaluations need to factor in potential alterations like tumor necrosis and host-mediated immune responses. Traditional caliper measurements of subcutaneous tumors, although commonly used, have their limitations - they cater predominantly to superficial tumors and are less accurate for tumors with irregular shapes. In addition, they cannot inform on the tumor composition such as the presence of necrosis.

Bioluminescence imaging (BLI) is a widely used functional imaging technique in the oncological field especially for assessing tumor growth and development, as well as for evaluating the efficacy of candidate therapeutics [2]. Relying on the expression of a light-emitting enzyme luciferase by implanted tumor cells and on the presence of a delivered chemical substrate luciferin [3], BLI captures emitted light, offering high sensitivity. However, it requires tumor cells engineering to stably express the firefly luciferase gene before engraftment into mice to form tumors. Its sensitivity, however, may be reduced for deeper structures due to light absorption and scattering by surrounding tissues [2, 4]. Monitoring bioluminescence demands animal handling as it requires injection of luciferin and anesthesia at each time-point, and there is a latency in signal acquisition, typically not reaching peak levels until at least 15 min post-luciferin administration. Meanwhile, high-frequency ultrasound (HFUS) offers real-time imaging, high spatial resolution, and cost-efficiency [5]. Notably, the prevailing body of research leverages ultrasound predominantly for cardiovascular studies [6]. When employed for tumor assessment, the prevalent experimental protocols documented in literature typically involve 3D imaging coupled with manual or semi-automated tumor segmentation. Although this enhances precision, it necessitates the use of anesthesia for mice and is not time-efficient, with scanning time of 10 min per animal in average [6, 7]. While BLI and HFUS both are capable of assessing tumor burden [4, 5, 8], they have distinct strengths and weaknesses.

Drawing inspiration from clinical tumor assessment protocols in humans, we have developed a fast-scan protocol for HFUS and conducted a thorough comparison with BLI for the assessment of breast tumor graft. To this aim, we utilized the E0771 cell line, originally derived from a spontaneous mammary gland tumor in C57BL/6 mice [9]. This cell line demonstrates high tumor engraftment rates when implanted orthotopically into the mammary fat pad and effectively replicates the tumor microenvironment in an immunocompetent host.

This study focuses on the longitudinal monitoring of breast tumor grafts at various stages, benchmarking our findings against histopathological analyses. Moreover, we delve into pivotal biological factors such as the extent of tumor necrosis and the properties of the tumor microenvironment, with a particular emphasis on the infiltration of immune cells.

## Methods

### Cancer Model and Animal Protocols

#### Engineering of a Bioluminescent Mammary Gland Tumor Cell Line

The medullary breast adenocarcinoma cell line originally isolated as a spontaneous tumor from C57BL/6 mouse was obtained from CH3 BioSystems LLC (Amherst, NY, USA). It was modified to stably express the Luciferase reporter gene. In brief, the coding sequence of the luciferase reporter gene luc2 (*Photinus pyralis*), which has been codon optimized for mammalian expression, was amplified by PCR from the pGL4.50[luc2/CMV/Hygro] plasmid template (Cat.# E1310, Promega, Madison, WA). Flanking XhoI restriction sites were added for the subcloning into the SalI site of the pLENTI PGK DEST Vector (Plasmid 9065, Addgene Cambridge, MA). Lentiviral particles were obtained in the HEK293T cell line and used to infect E0771 cells. Blasticidin (5 µg/ml) resistant cells were amplified and tested for luciferase expression and bioluminescence in vitro. Then cells were maintained as described in [10]. To control for luciferase expression, cells grown at 70–80% confluency were washed twice with PBS1X and scraped in 300 µl of lysis buffer (Tris HCl 50 mM, pH 7.4, with 150 mM NaCl, 1 mM EDTA, 1% Triton X-100, and 1X Complete protease inhibitor (Roche)). For Western blot analysis, nearly equal amounts of proteins (20 µg) were separated on 8–14% SDS–PAGE and transferred onto nitrocellulose membrane. Membrane was blocked with milk 3% in 1× PBS, Tween-20 0.1%, and incubated overnight at 4 °C with anti-Luciferase (sc-57,604; 1/1000, Santa Cruz Biotechnology) and (reprobed) with anti-actin, (A-1978; 1/1000, Sigma). Secondary horseradish peroxidase (HRP), conjugated anti-Mouse and anti-Rabbit antibodies were from Jackson ImmunoResearch. Signals were acquired using the (Amersham Imager 600).

#### In Vivo Studies

Mice breeding and maintenance were done in the accredited IGBMC/ICS animal house (C67-2018-37), in compliance with French and EU regulations on the use of laboratory animals for research. Animal experiments were approved by the ethical committee Com’Eth (Comité d’Ethique pour l’Expérimentation Animale, Strasbourg, France) and the French ministry of Higher Education and Research (#9177-2017030811336376v4). This project included the longitudinal evaluation of orthotopic tumor grafts in immunocompetent mice. In total we used 34 C57BL/6 mice aged from 18 to 24 weeks-old and evaluated tumor growth using 3 different modalities.

Mice were injected with 200,000 luciferase-expressing E0771 cells in the 4th left mammary gland at Day 0. The mice were then followed-up at Day 4, 7, 11, 14, 18 and 21. Depilation was done before injection and before each imaging session.

Mice were anesthetized using isoflurane. Induction was achieved with an isoflurane concentration of 3%, followed by maintenance at 1% in oxygen at a flow rate of 0.5-1 l/min.

All animals were euthanized at day 21 or when tumor longest diameter, assessed by caliper, was higher than 6 mm.

### Caliper Measurement

For each tumor, the longest diameter tumor L and its perpendicular diameter W were measured using a digital caliper. The tumor volume was estimated with the following formula:

$$Vo{l_{CAL}} = \frac{\pi }{6} \times L \times W \times \left( {L + W} \right)/2$$

### High-frequency Ultrasound Imaging Protocol

After caliper measurement, mice were scanned with hand-held high-frequency ultrasound probe (Vevo 3100, Fujifilm, VisualSonics). No anesthesia was required at that point.

Animal handling adhered to the National Centre for the Replacement, Refinement & Reduction of Animals in Research (NC3Rs) guidelines to ensure welfare-centric practices. Initially, the mouse was gently secured by holding the base of the tail with the thumb and forefinger, allowing it to stabilize itself using its forelimbs on a non-slip surface. Subsequently, a scruff restraint was employed to carefully expose the animal’s ventral side. While skin shaving was not required for ultrasound, it was needed for the BLI procedure. A layer of ultrasound gel, pre-warmed to 37 °C, was applied to the skin over the fourth left mammary gland to facilitate optimal acoustic contact. The ultrasound probe was then delicately positioned on the gland. All handling and ultrasonic scanning procedures could be performed by a single operator. The duration of each examination was calculated on images metadata, to assess efficiency of the procedure.

Tumor echogenicity was recorded (hyperechoic/isoechoic to the surrounding gland, slightly hypoechoic or strongly hypoechoic). Tumor heterogeneity was defined as areas of abnormal signal in the tumor, assessed semi-quantitatively as absent, minimal (< 10% of the tumor volume), significative (10–50%) or extensive (> 50%).

For tumor measurements, one image was taken in the plane of the longest diameter and another in the perpendicular plane. On each image, two perpendicular measurements were done, resulting in 4 measurements: L and W for tumor length and width, H1 and H2 for the measurements of tumor height in each of the two perpendicular images. Maximal subcutaneous tissue thickness between probe and tumor was also measured on the available images. To measure inter-reader variability, images from ten distinct individuals at different timepoints were independently evaluated by two readers of different level of experience. Finally, the tumor volume was estimated with the following formula (ellipsoid volume formula):

$$Vo{l_{US}} = \frac{\pi }{6} \times L \times W \times \left( {H1 + H2} \right)/2$$

Interval growth rate was defined as the change of volume between two consecutive timepoints normalized by the tumor volume at the first timepoint. For categorization purpose, stability was considered to be a change of less than 5%.

Growth pattern described the evolution of tumor volume over the entire follow-up period. The growth patterns were defined a posteriori, based on follow-up data clustering, as fast-growing, slow-growing, delayed-fast-growing and regressing tumors.

### Bioluminescence Imaging

Immediately after HFUS assessment, mice were injected intraperitoneally with 250 µL of D-Luciferin (15 mg/ml, XenoLight, Perkin Elmer). BLI scans were taken with a IVIS spectrum In Vivo Imaging System (PerkinElmer). For BLI acquisition, mice were anesthetized using 1.5–3% isoflurane.

With animals in dorsal decubitus, BLI images were sequentially acquired during 20 min, with an exposure time of 1 to 4 s, a field of view of 12.5 cm.

Analyses were conducted by capturing regions of interest (ROIs) from each image, from different timepoints after injection, quantifying the total flux and radiance, which represents the amount of light emitted per unit area and solid angle, as well as the variability of the signal and determination of the peak signal.

### Tumor Tissue Processing

After animal sacrifice, tumors were excised, their long axis measured and their weight was determined using a calibrated analytical balance, and reference tumor volume (Vol_REF_) was extrapolated from tissue mass assuming a density of 1 mg/mm3. Tumors were then fixed in formaldehyde and embedded in paraffin and cut.

### Hematoxylin and Eosin Staining and Tumor Cellularity Evaluation

Sections underwent hematoxylin and eosin (H&E) staining for assessment of general tumor structure, margins, necrosis and evaluation of tumor cellularity.

Evaluation of tumor cellularity has been described previously [11]. In our study, it involved the selection of a representative axial slice, tumor contouring and semi-automatic tumor cell counting using QuPath [12] algorithm. Tumor cells detection was based on the optical density using hematoxylin staining, with optimization of the sigma function and the minimum nuclear area, to reduce nuclear fragmentation and exclude small immune cells. The absolute number of tumor cells then normalized by the surface area of the contoured tumor to generate a tumor density. The tumor density was then multiplied by the surface area of the total tumor using the greatest linear dimension as the diameter.

These analyses were conducted on all tumors, except on fragmented, highly heterogeneous or irregular-shaped tumors, tumors with improper staining or other artifacts preventing a correct evaluation of tumor cellularity.

### Immunofluorescence Labeling

Sections also underwent immunofluorescence labeling targeting pancytokeratin, CD4, CD8 and CD34 for evaluation of the epithelial, immune and vascular components, respectively. Spatial distribution of markers and the number of positively stained cells per unit area were assessed.

### Statistical Analysis

Correlation analysis between imaging modalities, caliper and histological assessment, were conducted using Pearson test. For correlation between imaging (HFUS-derived tumor volume and BLI tumor brightness) and histology, imaging assessment was done the same day of mouse sacrifice. To assess inter-reader variability, two metrics were used: root mean square deviation (RMSD), that quantify the average magnitude of the difference between the measurements, and intraclass correlation coefficient (ICC) to assess the degree of agreement between readers.

Student test was used to compare quantitative values, such as the density of positively stained cells for immunofluorescence. A 2-tailed p-value of < 0.05 was considered statistically significant. Linear correlation was calculated with Pearson’s method (ρ).

The analyses utilized the following Python libraries: pandas (v.1.5.1), statsmodels (v.0.13.2) and matplotlib (v.3.5.1).

## Results

### Tumor Kinetics

**Fig. 1:**
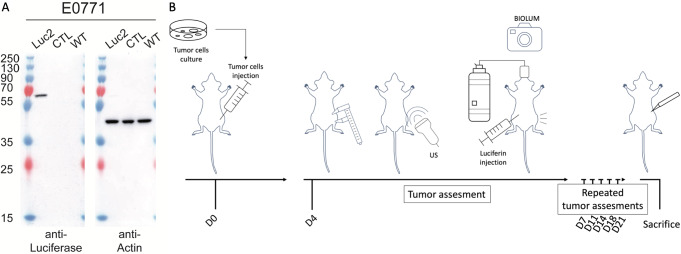
Bioluminescent tumor cells and follow-up protocol. **A**: Western blot analysis of Luciferase expression in whole cell protein extract (20 µg) of parental E0771 (WT), transduced with particles derived from the empty vector (CTL) and with particles derived from Luciferase containing vector (Luc2). The anti-Luciferase specific antibody recognized a single protein of around 62 KDa in Luc2 cells only. Anti-actin antibody was used as a loading control. **B**: Experiment workflow showing tumor implantation at day 0, tumor assessment by caliper, ultrasound and BLI (under anesthesia) at day 4,7,11,14,18 and 21

This research aimed to evaluate and compare different tumor monitoring methods in immunocompetent mice, focusing on caliper and ultrasound techniques, which do not require any modification of cancer cells, and on bioluminescence, which necessitates the expression of luciferase in tumor cells. To facilitate bioluminescence tracking, we engineered E0771 cells, compatible with the C57BL/6 mouse strain, to express luciferase via viral transduction followed by blasticidin selection. Western blot analysis confirmed luciferase expression in the modified E0771 cells (Fig. 1A). We then established a cohort of 34 mice, aged 18 to 24 weeks, for tumor implantation and monitored their progression until the designated endpoints of the experiment were met (Fig. 1B).

**Fig. 2:**
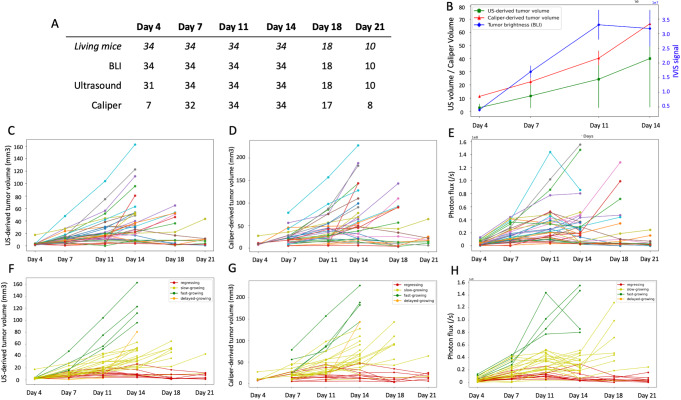
Assessment of tumor growth between the different modalities. **A** Table summarizing the presence of tumors detected by these different modalities, along with the corresponding number of living mice at each timepoint. **B** Mean tumor sizes for the period day 4 to day 14 (during that period, all mice are alive). **C**-**H**: Details of measurements for each mouse for ultrasound volumetry in mm^3^ (**C** and **F**), caliper-based volumetric estimation in mm^3^ (**D** and **G**) and tumor brightness (photons/sec) on BLI (**E** and **H**). In the graphs **C**-**E**, each color represents a mouse, while in graphs **F**-**H**, mice are grouped by their tumor growth pattern based on ultrasound volumetry (fast-growing, slow-growing, delayed fast-growing, regressing)

Following tumor cell implantation in the mammary gland, all the 34 mice of the study cohort developed tumors. However, tumor growth was not homogeneous. Based on the ultrasound volumetric assessment, we could distinguish four overall patterns of growth: fast-growing (*n* = 4), delayed-fast-growing (*n* = 1), slow-growing (*n* = 19), and regressing tumors (*n* = 10) (Fig. 2). At day 4 after injection, difference in the sensitivity of the 3 methods was noted. Only 20.6% of the tumors were palpable and measurable by caliper, this proportion sharply raised to 94% at day 7 (Fig. 2A). By contrast, by using bioluminescence monitoring, at day 4, all tumors emitted significantly more light signal than background, while by using ultrasound all tumors but 3 were seen. By day 7, the tumors were detected by palpation in the entire cohort, and all tumors were visible by ultrasound and BLI. From day 4 to day 11, most of the tumors presented with positive growth rates (Fig. 2C-E). Of interest, from day 11 to day 14 while both ultrasound and caliper metrics indicated linear tumor growth, there was a notable stabilization of the tumor signal as measured by BLI for several animals (Fig. 2C-E). A number of mice were sacrificed after day 14 and 18 as their tumor size reached the experiment terminal endpoint. The remaining mice were euthanatized at day 21.

**Fig. 3:**
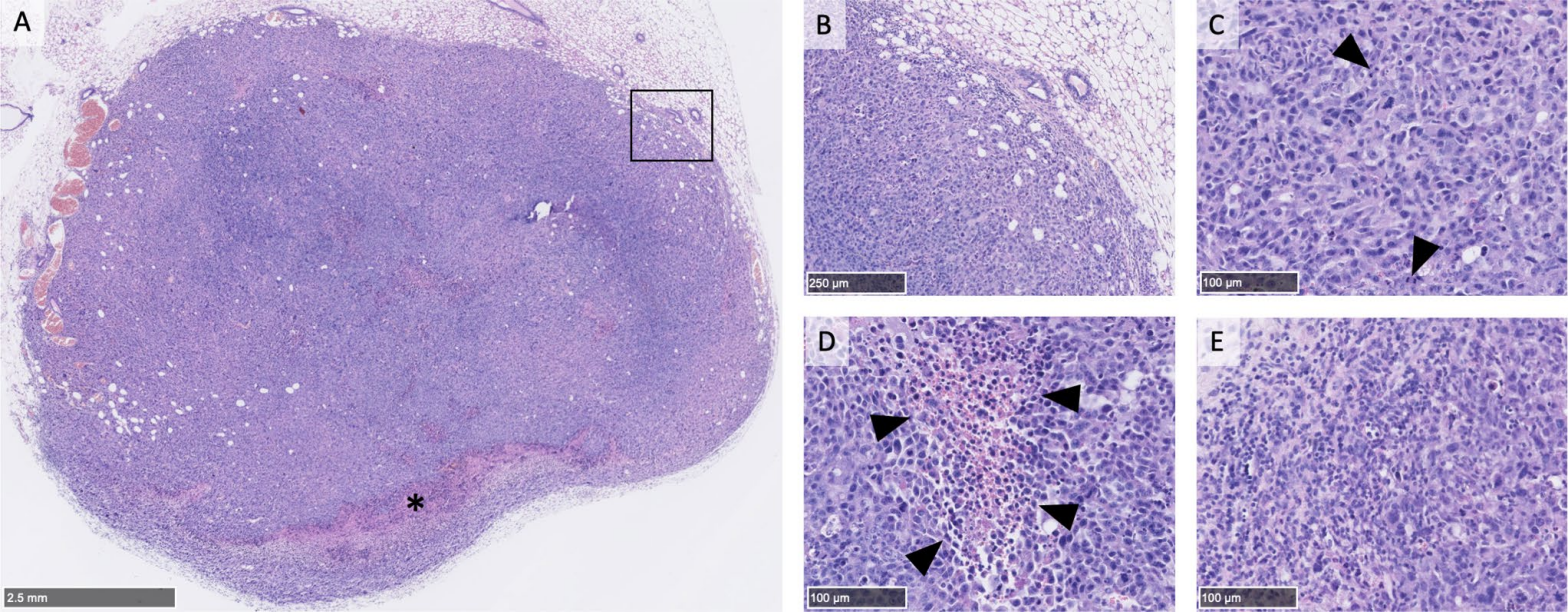
Representative hematoxylin and eosin-stained sections of a breast tumor graft. **A**: Low magnification reveals an oval-shaped tumor with limited necrosis (indicated by a star). The tumor margins are predominantly pushing with some entrapped adipocytes visible at the periphery. **B**: Medium magnification (10x) of the area outlined by a rectangle in A, highlighting the well-defined interface between the tumor and adjacent glandular tissue. The margin delineation suggests a pushing border rather than invasive growth. **C**: Higher magnification (25x) within the tumor core illustrates marked pleomorphism with numerous mitotic figures (denoted by arrowheads). The pleomorphism is characterized by variation in nuclear size and shape, as well as irregular chromatin patterns. **D**: A focused view (25x) of a necrotic region shows areas of coagulative necrosis (arrowheads), identifiable by pyknosis, characterized by densely stained, shrunken nuclei indicative of irreversible cell death. **E**: An additional high-power field (25x) in the tumor core displays infiltration by numerous small, round cells with hyperchromatic nuclei, consistent with lymphocytes. The distribution of these cells within the tumor stroma may suggest an immune response to the tumor

Histological examination of tumors collected either during the course of the experiment when reaching the terminal endpoint or at the end of the experiment, showed that they exhibited high cell density with marked atypia, lacking organized structural differentiation and demonstrating pushing margins. There was conspicuous pleomorphism and a high frequency of mitotic activity. The tumor microenvironment predominantly consisted of immune cells, including macrophages and a variable lymphocytic infiltrate. Fibrocytic areas were primarily confined to regions exhibiting coagulative necrosis. Figure 3 shows an example of the morphology and histology features of a typical tumor analyzed at the end of the experiment.

Collectively, these results show that the three methods enable monitoring tumor growth for different growth patterns, but they show differences in terms of sensitivity.

### High-frequency Ultrasound Reveals Early and Smaller Tumors and is more Accurate than Caliper

We then compared tumor growth measurements obtained by caliper and HFUS. All tumors were visible by HFUS at any timepoint, except at day 4, where 3 tumors were not clearly seen. At day 4, the largest axis measured by HFUS was 2.2 mm on average (± 0.7) and the average subcutaneous tissue thickness between probe and tumor was 6 mm (± 0.3).

**Fig. 4:**
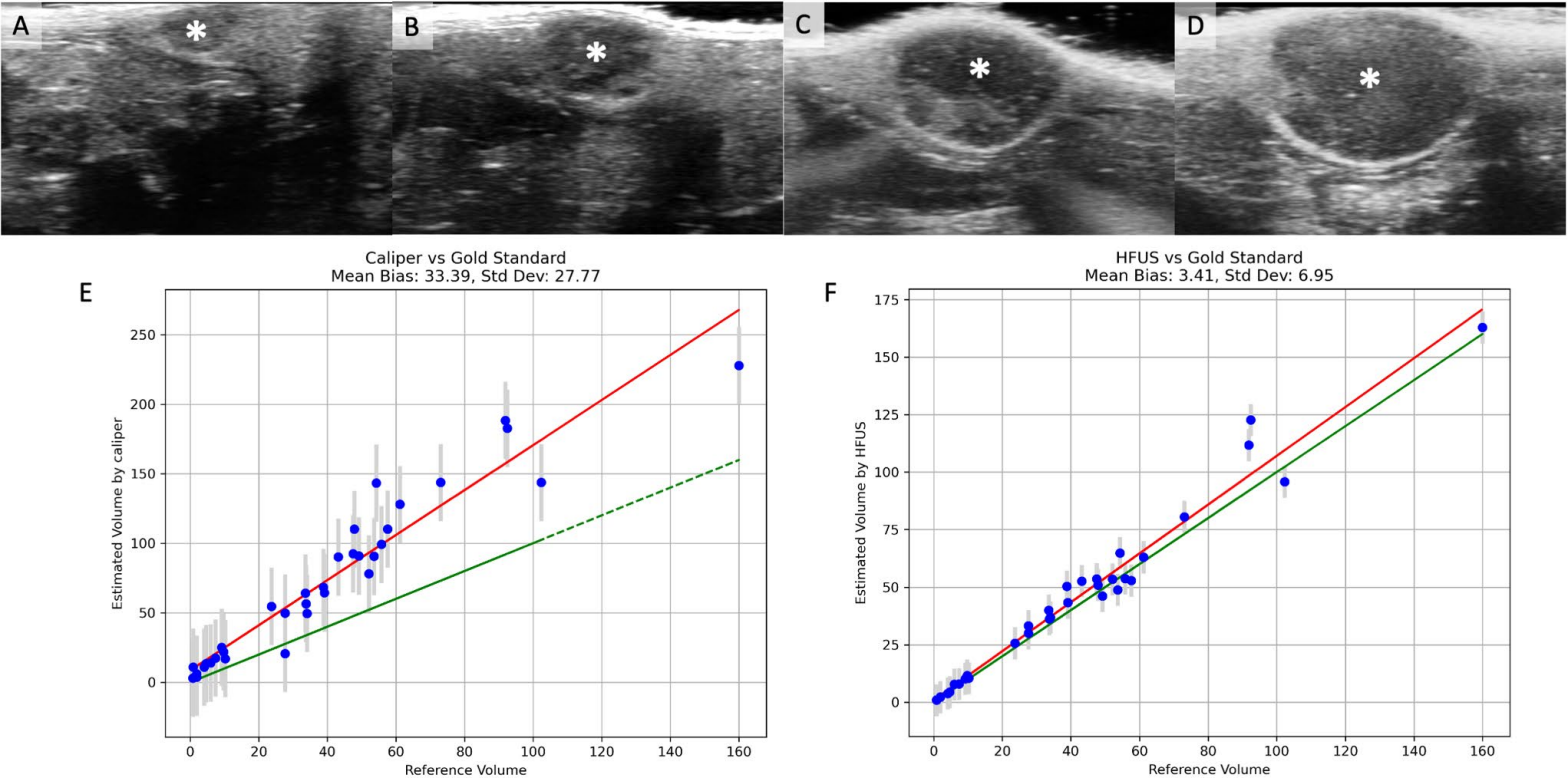
An example of longitudinal assessment of tumor growth on HFUS and comparison between volumetry obtained by HFUS/caliper with the volume obtained by the reference method. **A**-**D**: Longitudinal assessment of a typical tumor at day 4 (**A**), 7 (**B**), 11 (**C**) and 14 (**D**) showing a well-demarcated oval-shaped heterogeneous mass (asterisk). **E**: Correlation, for the entire cohort, between caliper-derived volumetric estimation (mm^3^) and the reference volume. **F**: Correlation, for the entire cohort, between HFUS-derived volumetric estimation (mm^3^) and the reference volume. Green line: line of equality, red line: best fit line. Std Dev: standard deviation

Based on images metadata, the average duration for the ultrasound scanning was brief, lasting only 47 s in average (± 18 s). All along the longitudinal evaluation, tumors were seen by HFUS as oval-shaped or polycyclic masses, with either circumscribed or microlobulated margins, and minimal to moderate internal heterogeneity (Fig. 4A-D).

At the end of the experiment, tumor volumes obtained by caliper and HFUS were compared with the reference tumor volumes measured after dissection (Fig. 4E-F). Tumor volume assessed by HFUS was more accurate than tumor volume assessed by caliper. The average bias – difference between the estimated volume (Vol_US_ or Vol_CAL_) and the volume of reference (Vol_REF_) – was 33.4 mm^3^ for caliper-based measurements and 3.41 mm^3^ for HFUS-based measurements (Fig. 4E-F). The assessment of inter-reader variability for diameter measurements in a subset of HFUS images revealed a RMSD of 1.47 mm. Furthermore, the ICC was 0.932, indicating a strong consistency between readers.

**Fig. 5:**
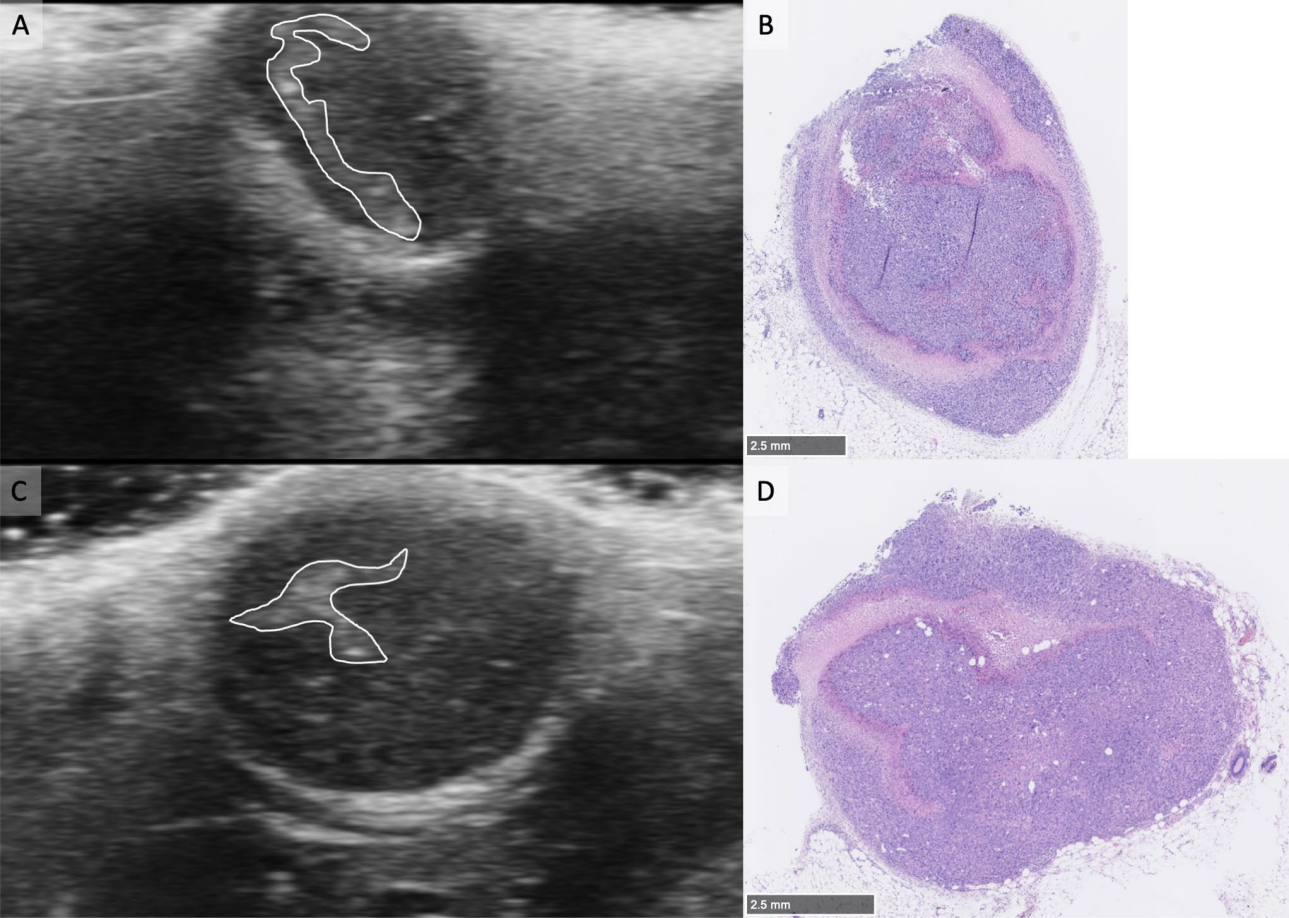
Tumor image comparisons between HFUS and histology. **A** and **C**: Typical images of ultrasounds obtained with two tumors. Within the tumor mass the presence of hyperechoic region is delineated in white. **B** and **D**: Matching histological HE images at low magnification, necrotic areas appear in pink

Of interest, HFUS could inform on the inner tumor composition. Partial tumor necrosis was confirmed by pathology in 12 tumors (minimal in 7 cases, significative in 5 cases): in all cases, necrosis was visible on the corresponding HFUS image as irregular areas of increased echogenicity inside the tumor (Fig. 5).

These results show that HFUS is more sensitive for smaller tumors, and the volume obtained by HFUS more accurate than caliper when compared to reference. Additionally, HFUS is able to assess tumor inner structure, in particular the presence of necrosis.

### Bioluminescence Imaging is Correlated to HFUS-Derived Tumor Volume and to Tumor Cellularity

**Fig. 6:**
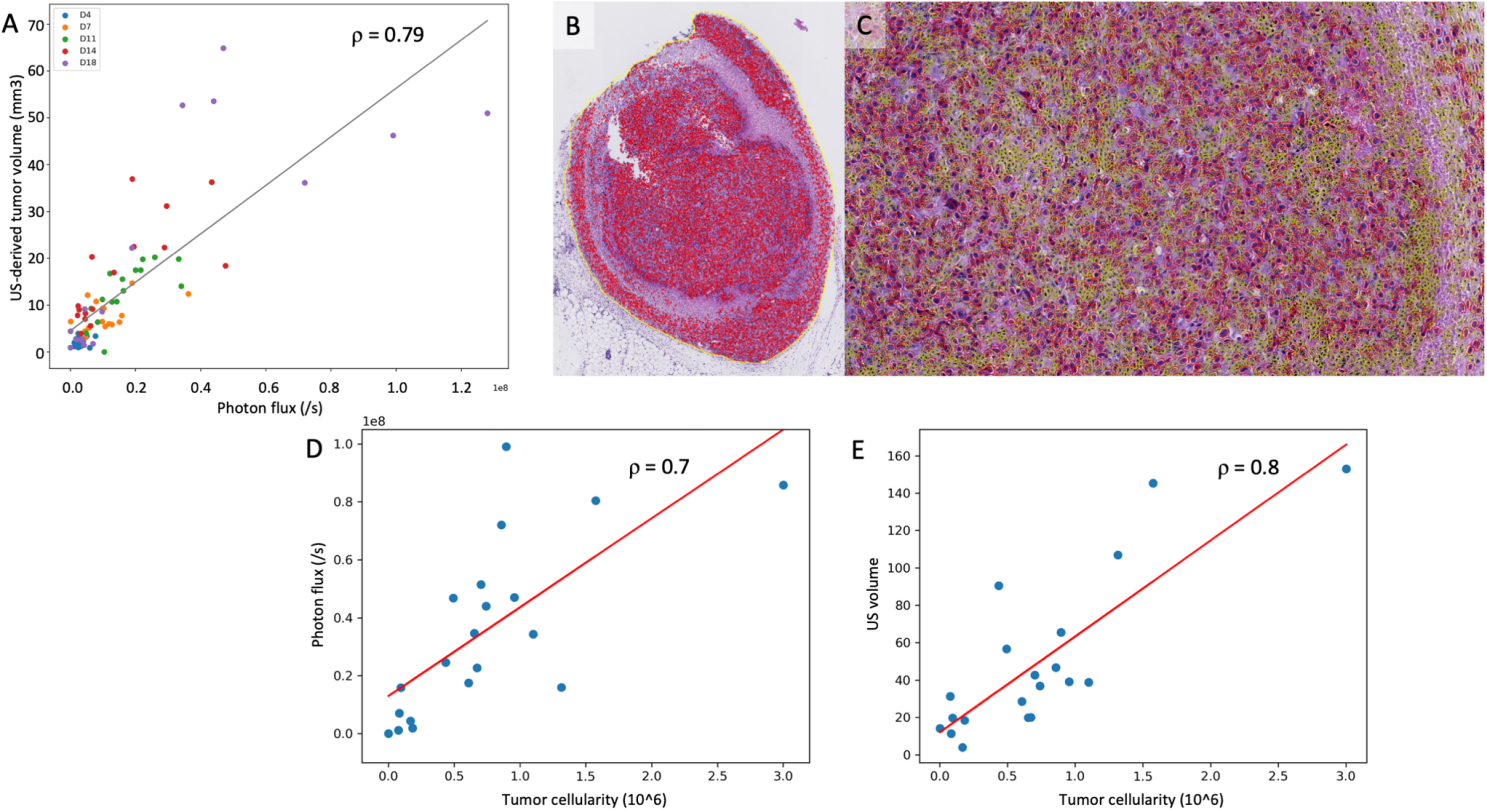
Comparison between tumor brightness on BLI, tumor volume on HFUS and tumor cellularity on histology. **A**: Correlation of tumor brightness on BLI and HFUS-derived tumor volume, for all measurements made during the follow-up period. Overall Pearson correlation coefficient is 0.79. **B**-**C**: Example of tumor cellularity assessment on hematoxylin-eosin-stained slice. First, tumor is delineated on low magnification (yellow outline), and a semi-automatic tumor cell counting using QuPath (delineated in red). The result of the segmentation is shown in **B** at low magnification. A 10X magnification is provided in **C**, with the additional segmentation of non-tumor cells in yellow. **D**-**E**: Correlation between tumor brightness in photon/sec **D** or ultrasound-derived volume **E** and tumor cellularity for all resected tumors. Each blue datapoint represents a resected tumor that has undergone tumor cellularity histological assessment and imaging assessment just on the day of the sacrifice

Having established that HFUS is more accurate to follow tumor growth, we then compared HFUS with BLI measurements. BLI acquisitions showed a positive signal in the tumor injected areas in all animals except for one animal at day 4. This indicates that all mice were properly injected with cancer cells. Considering all timepoints, there was a strong positive linear correlation between HFUS-derived tumor volume and tumor brightness on BLI (ρ = 0.79) (Fig. 6A). Correlation strength varied with time: Pearson correlation coefficient was 0.42 at day 4, 0.76 at day 7, 0.94 at day 11, 0.79 at day 14, 0.78 at day 18 and 0.88 at day 21.

After a last measurement, all tumors were removed, their morphology was assessed by hematoxylin-eosin staining and a semi-automatic assessment of their cellularity was conducted (Fig. 6B-C). We compared the cellularity of the tumors with the final tumor volume and the signal intensity measured by HFUS and BLI, respectively. As shown in Fig. 6D-E there is a strong correlation between cellularity and either tumor volume measured with HFUS (ρ = 0.8) or tumor brightness assessed on BLI (ρ = 0.7). Taken together these data indicate that both BLI and HFUS modalities provide consistent measures of tumor growth.

### Discrepancies of Tumor Growth Rate Between Modalities are Related to Tumor Infiltration by Immune Cells.

**Fig. 7:**
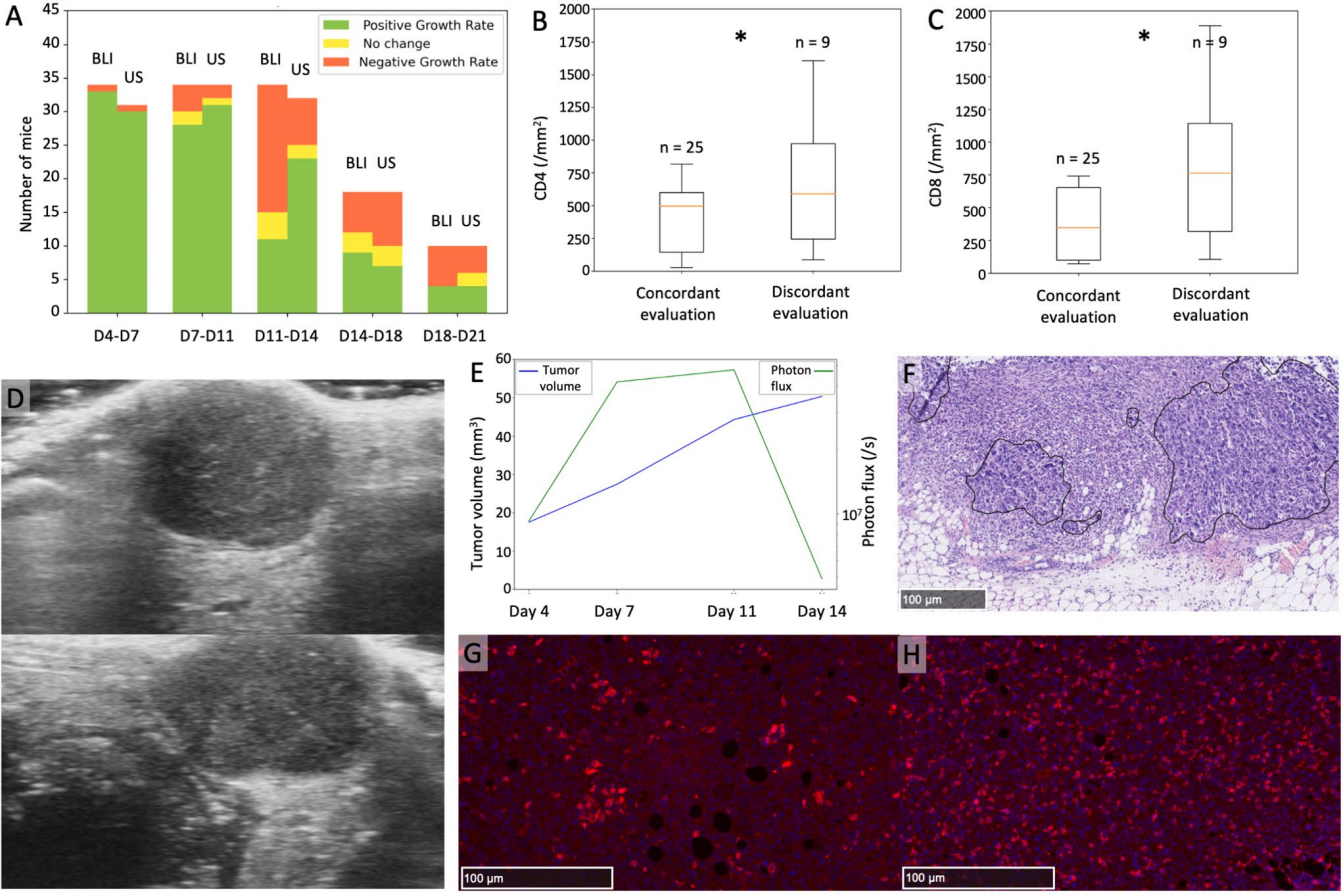
Comparison of tumor growth trends between HFUS and BLI and correlation with immune infiltration. **A**: For each time interval (x axis), the number of tumors presenting with increase (green), decrease (orange) or stability (yellow) is represented as a histogram. Discordances between BLI and HFUS are higher during the day 11 – day 14 interval. **B**-**C**: Tumor infiltration by CD4+ B and CD8 + cells C, measured by the number of IF-positive cells per mm^2^ on a representative section, is higher when imaging assessment is discordant. **D**-**H**: Example of a tumor with decreasing brightness on BLI but persistent tumor growth on HFUS, associated with diffuse lymphocytic infiltration. Ultrasound images showing a slightly heterogeneous tumor on two orthogonal plans **D**. Longitudinal assessment of the tumor **E** shows a pronounced decrease of tumor brightness from day 11 (green line) but a steady increase of tumor volume measured by HFUS (blue line). **F**: HE stained section shows the presence of cancer cell clusters (delineated with a black line) and immune cells. **G** and **H**: Immunofluorescence analyses to detect CD4 and CD8 cells show the presence of infiltrating T lymphocytes next to tumor cell clusters

Tumor growth rate observed with HFUS largely paralleled those seen with BLI. Typically, when observed individually, an increase in tumor volume on HFUS corresponded to an increased brightness on BLI. However, there was a notable spike in discrepancies between the two methods during the day 11 to day 14 interval (Fig. 7A).

Given that ultrasound measures tumor volume, while BLI measures an enzymatic activity of cancer cells, we reasoned that the discrepancies could be attributed to the presence of non-tumor cells from the tumor microenvironment and/or necrosis. To address this possibility, we made immunofluorescent staining to mark immune cells. When we examined the histology of individual tumors with discordant measurements, we found a pronounced immune infiltration. We then compared the extent of immune infiltration among consistent and disagreeing measurements and found an association of the discordant individual tumor with the presence of an immune infiltration (*p* = 0.04 for CD8 infiltration, *p* = 0.05 for CD4 infiltration) (Fig. 7B-C). Interestingly, when assessing vascularization, no significant differences emerged between the two groups. Of note, the necrosis ratio tended also to be higher in discordant cases (*p* = 0.05). Figure 7D-H shows an example of a tumor trajectory that gave non-consistent results between HFUS and BLI, associated with the presence of diffuse lymphocytic infiltration. Tumor growth kinetics assessed by HFUS showed a steady increase (Fig. 7A-B), in contrast a sharp decrease of BLI signal was observed at day 11. Histology of that tumor at the end point revealed the presence of immune infiltration by T lymphocytes around clusters of cancer cells (Fig. 7F-H).

**Fig. 8:**
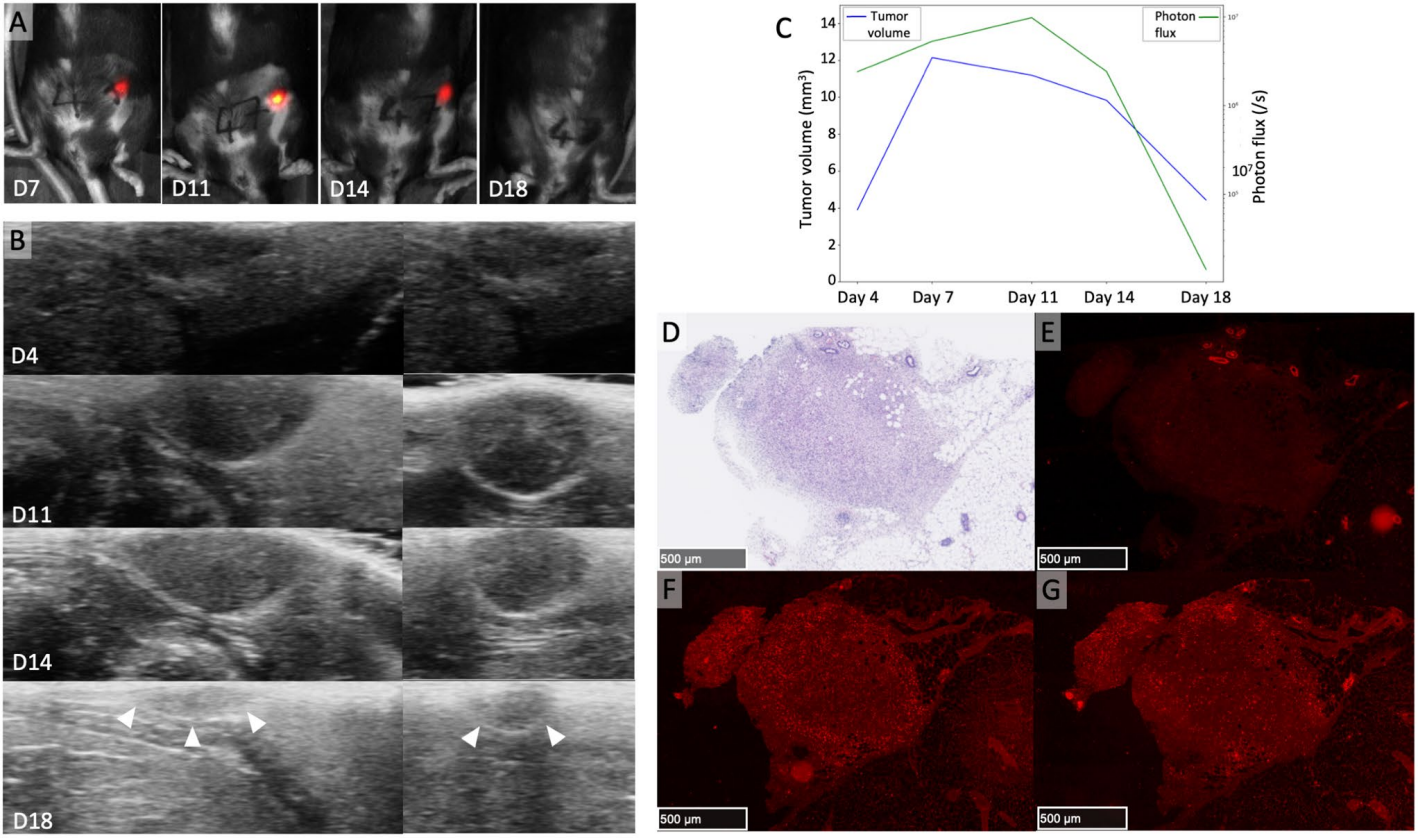
Tumor dynamics can be followed by HFUS and BLI. **A**: Tumor growth measured by BLI from day 7 to 11 and decreases from day 11 to 14, no detectable signal at day 18. **B**: Parallel images obtained by HFUS, images on the left and on the right correspond to the two orthogonal plans, tumor growth is noticeable until day 14 and then a rapid diminution of the tumor size is observed from day 14: at day 18, the tumor is barely distinguishable from the surrounding tissue due to its high echogenicity. **C**: Tumor growth kinetics showing an increase and a decrease of tumor brightness (green line) paralleled by tumor volumes measurements by HFUS (blue line). **D**-**G**: HE stained slice at low magnification **D** centered on the tumor bed showing edematous and inflammatory changes with no residual epithelial tumor cells (**E**: immunofluorescence for pancytokeratin) but intense lymphocytic infiltration (**F**: immunofluorescence for CD8 and **G**: immunofluorescence for CD4)

One unique case of complete tumor regression was observed on histology. As depicted in the Fig. 8, for this case, from day 7 onward, HFUS demonstrated a gradual decrease in tumor volume, and revealed a markedly hyperechoic structure strongly indicative of a non-viable tumor. A concurrent decrease in tumor brightness on BLI was noted, especially post day 11. Histology on the residual tumor tissue showed an intense inflammatory infiltration and the absence of keratin-positive cancer cells.

These results support the notion that inconsistent results observed while monitoring tumor growth by HFUS and BLI are linked with the presence of immune infiltration and/or necrosis. Moreover, HFUS may provide functional insights into the tumor biology through tumor echogenicity.

## Discussion

Our investigation has highlighted the potential of fast ultrasound scan as a reliable modality for the longitudinal assessment of xenografted tumors. Notably, HFUS was able to detect small breast tumor (2.2 mm in average diameter) from as early as day 4 post-implantation. Moreover, this study shows that BLI and HFUS provide complementary information on the biology of the tumor.

Tumor growth measurement with caliper is the predominant method used in preclinical studies. Indeed caliper measurements are used in 88.4% of the studies reported in the literature [13]. Caliper has some advantages, its simplicity, it does not require cancer cells alteration nor anesthesia and is cost-effective. However, this method has limitations, it requires the tumor to be palpable and is not very precise. In particular, by omitting tumor height, an important morphologic information is lost. While the average height/width ratio has been found to be 1/3, caliper measurements lead to a systematic overestimation of tumor volume [14]. Our study indicates that HFUS significantly outperforms caliper measurements by providing a comprehensive view of the tumor, thus avoiding the common overestimation of tumor volume associated with caliper use. Importantly, HFUS is more sensitive than caliper to detect early tumors, as mentioned in the literature HFUS can detect very small tumors, depending on conditions and equipment, potentially recognizing lesions as minute as 0.25 mm [15].

In our study, the consistency of diameter measurements in acquired HFUS images was excellent. However, discrepancies between the estimated HFUS-derived tumor volume and the actual tumor volume can occur due to several factors. The selection of imaging planes in HFUS is operator-dependent, which it is why we recommend having the same operator conduct the ultrasound assessments consistently over time. Additionally, discrepancies can arise from irregularities in tumor geometry or non-spherical growth patterns, as tumor volume estimations are typically based on an ellipsoid formula. These issues are more pronounced in endogenous tumor models than in tumor grafts, where growth patterns tend to be more uniform [16, 17].

We established a fast scan HFUS protocol, lasting less than 60 s per animal, which does not require anesthesia and once equipped is time and cost-effective. An added value of this protocol is its accordance with the 3R principle, aiming to refine research methods to cause less harm and distress to animals, reduce the number of animals used, and replace animals with alternative techniques whenever possible. By delivering precise and consistent volumetric data, HFUS reduces the need for larger sample sizes by enhancing the quality of data obtained from each animal. Another value noted was that HFUS can provide insight into the tumor biology since subtle difference in echogenicity are associated with the presence of necrotic region.

While BLI remains a sensitive tool for tracking cellular dynamics in vivo, our study established that there is a robust correlation between HFUS volumetric measurements and BLI luminosity. Although HFUS alone may serve as a sufficient imaging technique for tumor volume assessment over time, BLI is a valuable preclinical tool for tracking cell populations in vivo due to its high sensitivity and to the fact it records only active and living cells. However BLI faces some challenges, accurate quantification is compounded by light scattering, low emitted light intensity, and temporal and dose-dependent variability post-luciferin injection [3, 18]. Moreover, the use of bioluminescent cancer cells may introduce variability related to luciferase protein levels or bioluminescent activity, presenting an additional layer of experimental bias [19]. Finally, BLI necessitates anesthesia.

The biological pertinence of HFUS is reinforced by its significant correlation with tumor cellularity determined by histopathological analysis, a crucial prognostic indicator and a benchmark for gauging therapeutic efficacy [20, 21].

Recognizing an intramammary tumor graft using high-frequency ultrasound is typically straightforward due to the hypoechogenicity of tumors compared to normal breast tissue. However, we acknowledge challenges in detecting very small tumors. Additionally, while mammary lymph nodes are usually identifiable by their fatty hilum, their absence can make them resemble small tumors [22]. Continuous refinement of the HFUS technique could further minimize these limitations.

In our study, we observed that in the interval between days 11 and 14, a notable proportion of mice presented with stabilization of the BLI signal. This plateau in BLI can be attributed to several biological processes, including inadequate vascularization of the tumor [4, 23]. It is important to highlight, however, that this observed stabilization in BLI was not consistently mirrored by a corresponding stabilization in tumor volume when measured with HFUS. Our observations indicated discrepancies in tumor growth assessment when comparing BLI with HFUS during that period of time, which appeared to correlate with an elevated rate of necrosis and increased lymphocyte infiltration. Our findings suggest that BLI and HFUS assessment may diverge in specific model or at some stage of cancer progression, because they capture different biological processes, highlighting the complementarity of these techniques.

HFUS extends its utility beyond mere volumetric analysis. Its capacity to delineate tumor heterogeneity and visually identify regions of necrosis help delivering precious insights into treatment responses – for instance for tumor-vascular disrupting agents [24], thus complementing BLI data.

Our investigation encountered a few constraints. Primarily, to align with the principle of reducing animal use, subjects were euthanized according to a predetermined tumor volume threshold rather than at fixed intervals; consequently, we lack histological data and a benchmark for tumor volumes on days 4, 7, 11, and 14. Additionally, our study population exhibited a notable incidence of spontaneous tumor regression, either partial or complete. This phenomenon may be attributed to the immunocompetent host’s response to the tumor grafts, which, while insightful, could restrict the applicability of our findings across different experimental setups. Finally, we observed no evidence of distant organ metastasis among the tumors. While ultrasound serves as an effective tool for primary tumor evaluation, its capacity for detecting metastatic spread is limited due to the intricate and dispersed nature of metastases. Therefore, for a thorough investigation of potential metastatic dissemination, a more advanced and sensitive imaging technique would be preferable, such as Positron Emission Tomography (PET) imaging [25].

## Conclusion

A rapid high-frequency ultrasound protocol facilitates precise, longitudinal monitoring of tumor grafts, encompassing volume measurements, tumor cellularity, and necrosis evaluation. This non-invasive approach, requiring no injections and completed under a minute on awake animals, significantly minimizes distress, aligning with refined procedural standards and enhancing animal welfare.

## Data Availability

No datasets were generated or analysed during the current study.
